# Phenotypic Antimicrobial Resistance in *Escherichia coli* and *Salmonella* spp. Recovered from Broiler-Farm Environmental Matrices in Morocco

**DOI:** 10.3390/antibiotics15080731

**Published:** 2026-07-28

**Authors:** Walid Atiki, Najia Ameur, Atika Zidouh, Abdelmoula Elouardi, Latifa Tahri

**Affiliations:** 1 GeoBiodiversity and Natural Patrimony Laboratory GEOPAC Research Center Scientific Institute, Mohammed V University in Rabat, Ibn Battuta Av, Rabat B.P. 1040, Morocco; 2 Department of Microbiology of Water, Food and Environment, National Institute of Hygiene, BP: 769, Agdal, 27, Avenue Ibn Batouta, Rabat 10000, Morocco; 3 Laboratory of Natural Resources and Sustainable Development, Department of Biology, Faculty of Sciences, Ibn Tofail University, Kenitra 14000, Morocco

**Keywords:** antimicrobial resistance, multidrug resistance, *Escherichia coli*, *Salmonella*, broiler farms, poultry environment, One Health, Morocco, environmental surveillance

## Abstract

**Background/Objectives**: Antimicrobial resistance (AMR) in poultry-production environments is an important One Health concern. This phenotypic baseline study investigated the detection and antimicrobial-resistance profiles of *Escherichia coli* and *Salmonella* spp. recovered from broiler-farm environmental matrices in the Rabat–Salé–Kénitra region of Morocco. **Methods**: A cross-sectional field study was conducted in 13 broiler farms on 35 sampling dates between 24 November 2023 and 28 April 2025. A total of 543 samples were collected from poultry manure, drinking troughs, farm soil, soils approximately 200 and 500 m from the poultry houses, and well water. Isolates were identified by culture and API 20E; *Salmonella* spp. identification was additionally confirmed by real-time PCR. Disk-diffusion results were interpreted using applicable CLSI M100, 31st edition, criteria, and colistin was tested by the colistin broth disk elution MIC method. Multidrug resistance (MDR) was defined as resistance to at least one agent in three or more antimicrobial classes. **Results**: *Escherichia coli* and *Salmonella* spp. were detected in 190/543 (35.0%) and 127/543 (23.4%) samples, respectively. Among *E. coli* isolates, 115/190 (60.5%) were MDR; the highest matrix-specific proportions occurred in farm soil (85.7%), drinking troughs (83.3%), and poultry manure (75.7%). Among *Salmonella* spp. isolates, 82/127 (64.6%) were MDR, with the highest proportions in poultry manure (92.9%) and drinking troughs (78.3%). The predominant resistance rates were ampicillin (84.7%), tetracycline (61.6%), and chloramphenicol (55.8%) in *E. coli*, and nalidixic acid (73.2%), ampicillin (61.4%), and tetracycline (55.9%) in *Salmonella* spp. Resistant isolates were also detected in surrounding soils and well water; however, the study design did not permit source attribution or inference of transmission direction. **Conclusions**: Broiler-farm environmental matrices in the study area may represent reservoirs of phenotypically antimicrobial-resistant *E. coli* and *Salmonella* spp. The findings provide baseline evidence supporting integrated One Health surveillance, improved biosecurity, manure and litter management, water-system sanitation, and responsible veterinary antimicrobial use. Genomic typing and environmental source-tracking are required to determine persistence and transmission pathways.

## 1. Introduction

Poultry production contributes substantially to food security, nutrition, and rural economies by supplying meat and eggs. However, intensified production also creates challenges related to flock health, bacterial contamination, and food safety. *Salmonella* spp. and *Escherichia coli* are frequently encountered along the poultry-production continuum and are important because they can compromise animal health, food safety, and public health [1,2,3,4,5].

Antimicrobial resistance (AMR) is a major threat to animal and public health and reflects interconnected selection pressures across human medicine, livestock production, and the environment [6]. Antibiotic growth promoters historically improved feed conversion, growth performance, and disease control in intensive broiler systems, contributing to their prolonged use in poultry production [7]. International surveillance frameworks, including those coordinated by the World Organisation for Animal Health, emphasize the contribution of veterinary antimicrobial use to the broader resistance-selection landscape [8,9]. Monitoring poultry-associated *E. coli* and *Salmonella* spp. is therefore important for One Health AMR surveillance [9,10].

The poultry-farm environment is an interface at which animals, workers, water, soil, litter, and manure interact [11,12]. Feces, litter, manure handling, drainage, dust, and runoff have been proposed in the literature as possible routes by which bacteria may enter environmental matrices [13,14,15,16,17,18]. These routes are presented here as background hypotheses; the present study did not measure antimicrobial residues, quantify bacterial movement, or determine transmission pathways.

Environmental bacterial communities may also carry resistance genes on plasmids, integrons, transposons, and other mobile genetic elements [19,20,21,22]. Nevertheless, the present investigation assessed culture-based detection and phenotypic antimicrobial susceptibility only. Resistance genes, plasmids, integrons, horizontal gene transfer, and genetic relatedness were not directly investigated and are therefore not inferred from the results.

Accordingly, this study aimed to determine the detection rates of *E. coli* and *Salmonella* spp. in six broiler-farm environmental matrices and to characterize their phenotypic antimicrobial-resistance and MDR profiles. The work was designed as baseline environmental surveillance within a One Health framework rather than as a source-attribution or transmission study.

## 2. Results

### 2.1. Occurrence of Escherichia coli and Salmonella spp. In Broiler Farm Environmental Samples

A total of 543 environmental samples were analyzed for *Escherichia coli* and *Salmonella* spp. Overall, *E. coli* was detected in 190 samples (35.0%), whereas *Salmonella* spp. was detected in 127 samples (23.4%). For *E. coli*, the highest detection rates occurred in poultry manure (37/78; 47.4%), soil approximately 500 m from the farms (35/79; 44.3%), and farm soil (42/98; 42.9%). Lower rates were recorded in soil approximately 200 m from the farms (30/79; 38.0%), drinking troughs (24/79; 30.4%), and well water (22/130; 16.9%).

For *Salmonella* spp., the highest detection rates occurred in poultry manure (28/78; 35.9%), farm soil (34/98; 34.7%), and drinking troughs (23/79; 29.1%). Lower rates were found in soil approximately 200 m from the farms (14/79; 17.7%), well water (18/130; 13.8%), and soil approximately 500 m from the farms (10/79; 12.7%). These values describe detection within the sampled matrices and should not be interpreted as population prevalence. The complete matrix-specific detection results are summarized in Table 1.

### 2.2. Monthly Detection of Escherichia coli and Salmonella spp.

**Figure 1:**
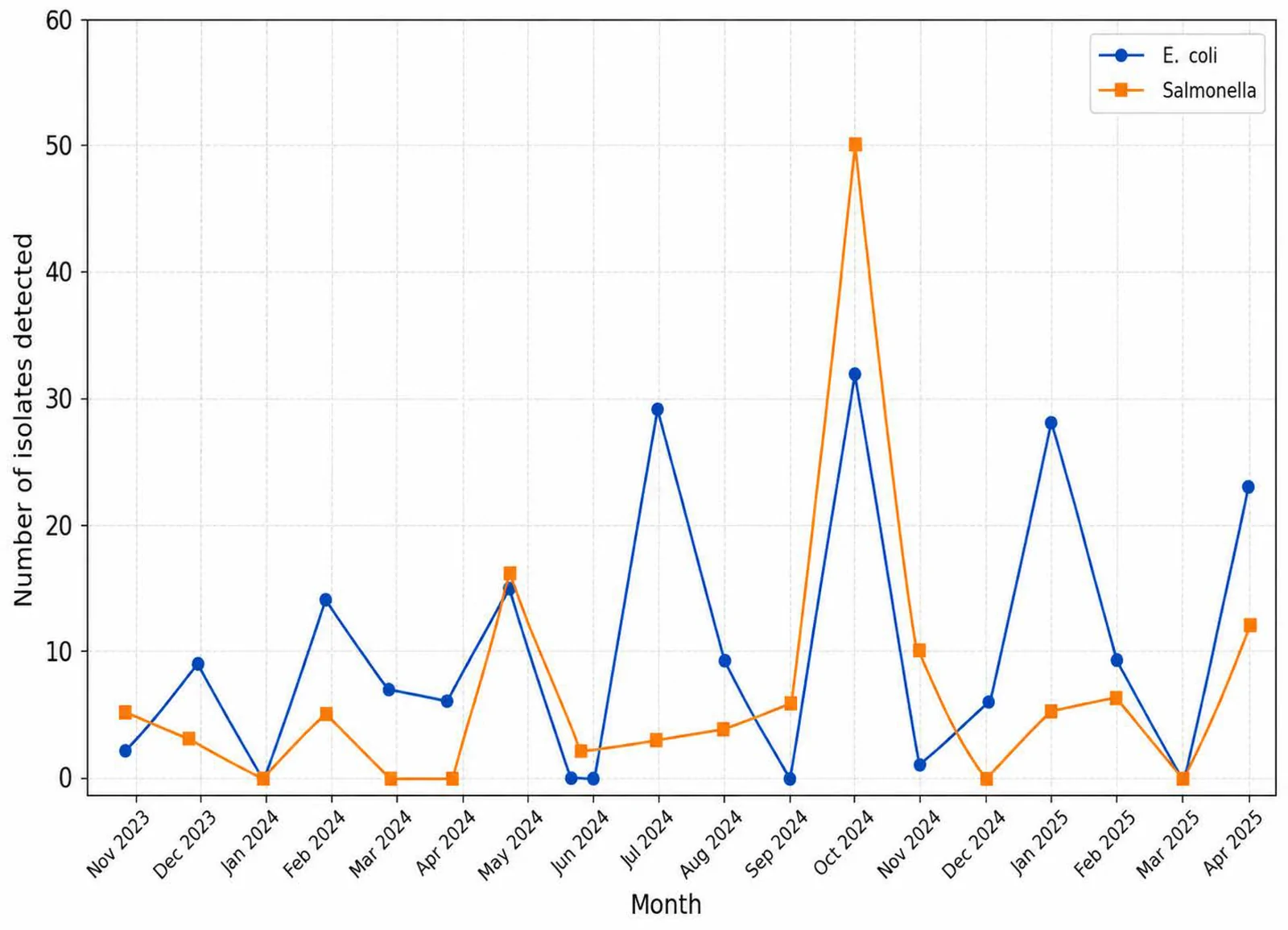
Monthly numbers of *Escherichia coli*- and *Salmonella* spp.-positive samples from November 2023 to April 2025. Monthly sample denominators are provided in Supplementary Table S1.

Monthly detections fluctuated across the sampling period and reflected an irregular field schedule rather than equally spaced monthly surveillance (Figure 1). *Escherichia coli* detections ranged from 0 to 32 per sampled month. The largest counts were recorded in October 2024 (32), July 2024 and January 2025 (28 each), and April 2025 (23). The corrected month-level counts and corresponding numbers of samples analyzed are provided in Supplementary Table S1.

*Salmonella* spp. detections ranged from 0 to 50 per sampled month. The highest count was observed in October 2024 (50), followed by May 2024 (13), April 2025 (12), and November 2024 (9). Four detections were recorded in December 2023. Months without sampling are not displayed as zero-detection months.

Because monthly sampling totals varied from 6 to 87 samples and the distribution of matrices also differed among months, these results are presented as descriptive detection counts rather than monthly prevalence. Month-by-matrix sample counts, positive detections, and isolate denominators are reported in Supplementary Table S1.

### 2.3. Geographical Distribution of Escherichia coli and Salmonella spp. Detections

**Figure 2:**
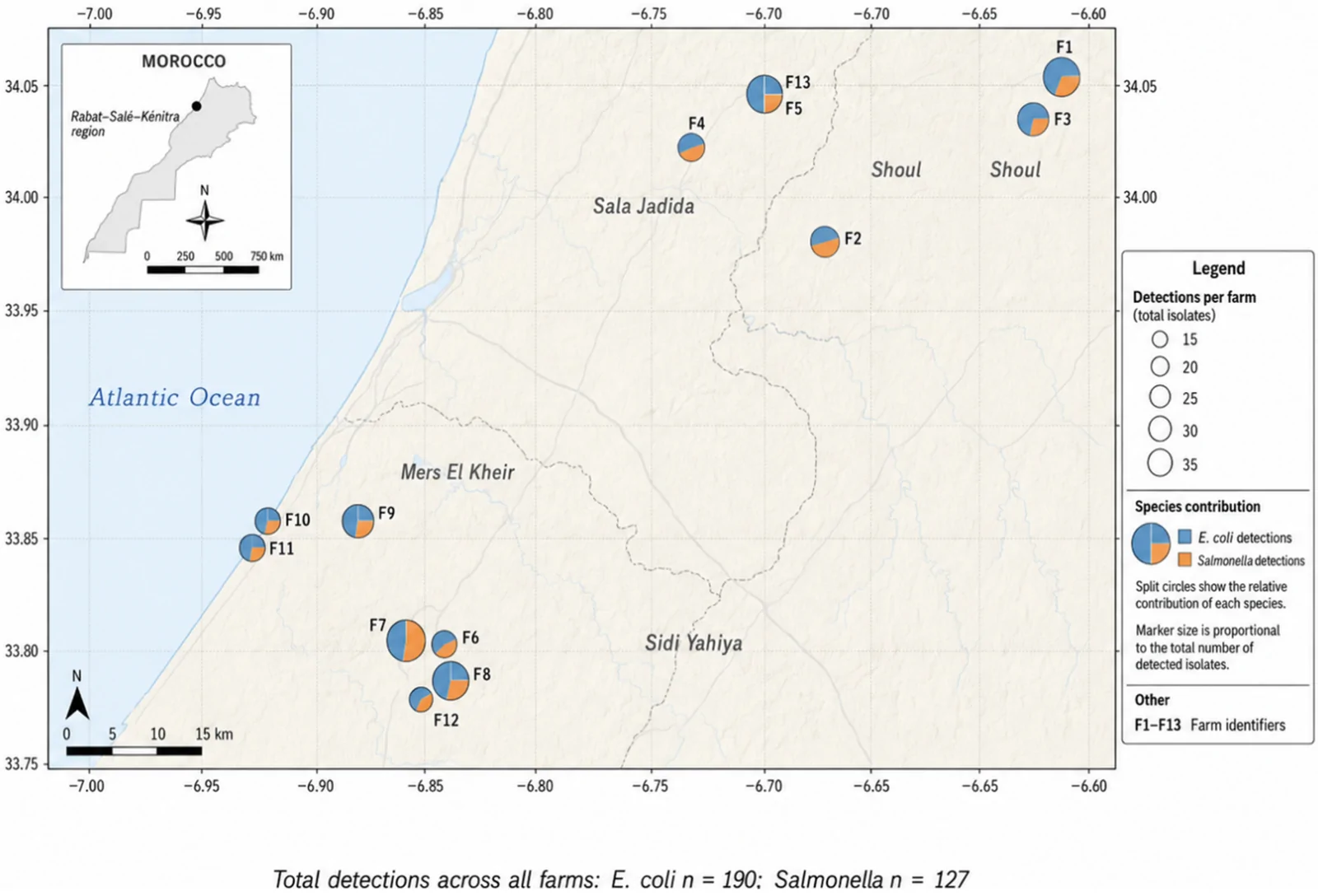
Geographical distribution of *Escherichia coli* and *Salmonella* spp. detections in broiler-farm environments in the Rabat–Salé–Kénitra region, Morocco. Circle size represents the total number of detections per farm, split-circle sectors show the relative contributions of the two bacterial groups, and F1–F13 identify the farms.

Both bacterial groups were detected in all four surveyed locations: Sidi Yahia, Mers El Kheir, Sala Jadida, and Shoul (Figure 2; Table 2). For *E. coli*, Sidi Yahia accounted for 61 detections (32.1% of *E. coli*-positive samples), Mers El Kheir and Sala Jadida for 45 each (23.7%), and Shoul for 39 (20.5%).

For *Salmonella* spp., Sidi Yahia accounted for 37 detections (29.1% of *Salmonella*-positive samples), Shoul for 31 (24.4%), Mers El Kheir for 30 (23.6%), and Sala Jadida for 29 (22.8%). These percentages describe the distribution of positive detections among the sampled locations and do not establish geographical risk differences because sampling intensity was not designed to be representative.

### 2.4. Phenotypic Antimicrobial Resistance Profiles of E. coli and Salmonella spp. Isolates

**Figure 3:**
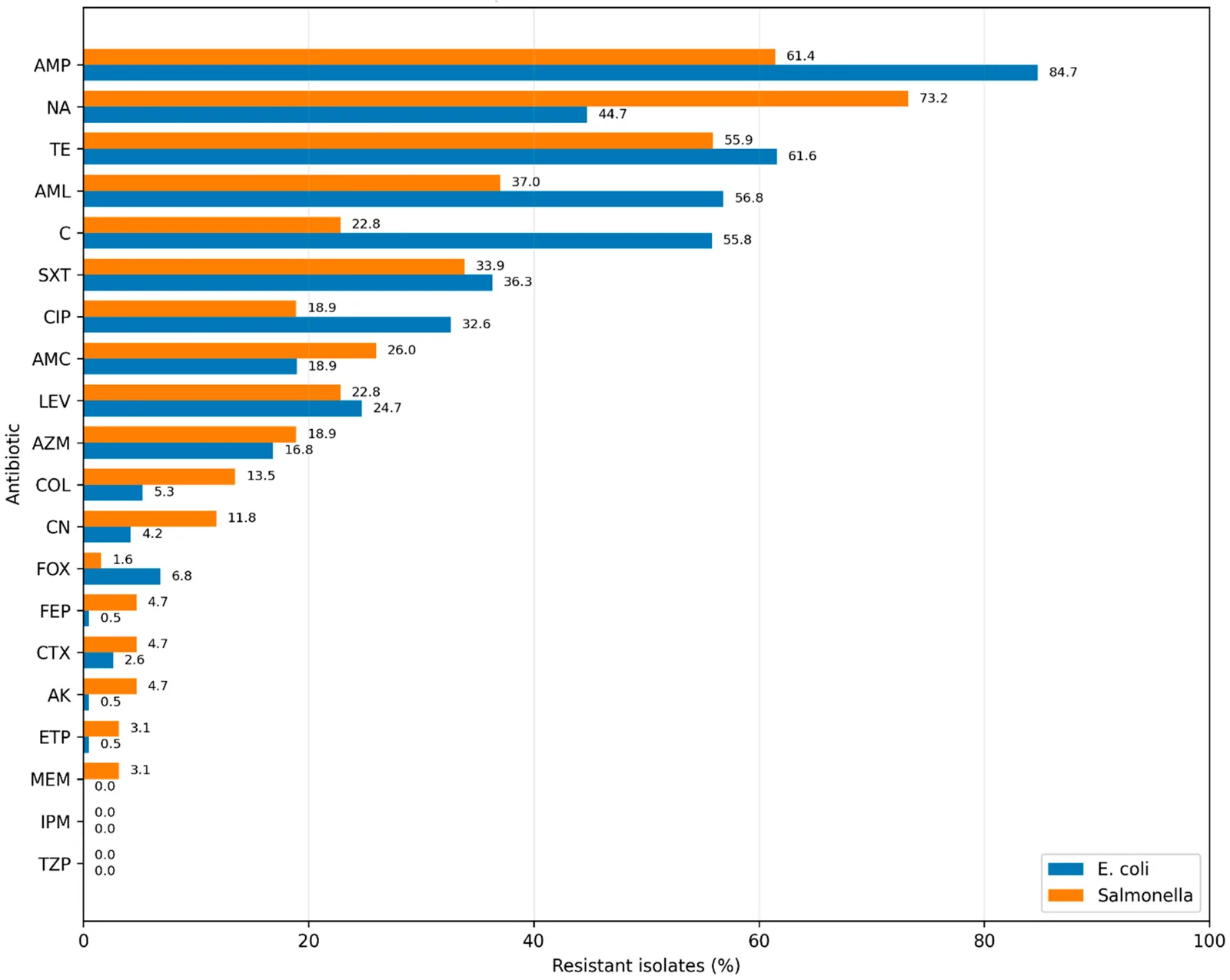
Phenotypic antimicrobial resistance among *Escherichia coli* (n = 190) and *Salmonella* spp. (n = 127) recovered from broiler-farm environmental samples. Colistin values are based on MIC testing. *Salmonella* levofloxacin disk results were not interpreted categorically. Agents lacking applicable general CLSI M100 criteria were excluded from the figure. AMP: ampicillin; AMC: amoxicillin–clavulanic acid; FOX: cefoxitin; CTX: cefotaxime; FEP: cefepime; TZP: piperacillin–tazobactam; CN: gentamicin; AK: amikacin; NA: nalidixic acid; CIP: ciprofloxacin; LEV: levofloxacin; SXT: trimethoprim–sulfamethoxazole; C: chloramphenicol; TE: tetracycline; COL: colistin; IPM: imipenem; ETP: ertapenem; MEM: meropenem.

Using antimicrobial agents with applicable categorical criteria, *E. coli* showed the highest resistance to ampicillin (161/190; 84.7%), tetracycline (117/190; 61.6%), chloramphenicol (106/190; 55.8%), nalidixic acid (85/190; 44.7%), trimethoprim–sulfamethoxazole (69/190; 36.3%), and ciprofloxacin (62/190; 32.6%) (Figure 3). Lower rates were observed for levofloxacin (24.7%), amoxicillin–clavulanic acid (18.9%), cefoxitin (6.8%), colistin by MIC (5.3%), gentamicin (4.2%), cefotaxime (2.6%), cefepime (0.5%), amikacin (0.5%), and ertapenem (0.5%). No resistance was detected to imipenem, meropenem, or piperacillin–tazobactam.

Among *Salmonella* spp., the highest resistance rates were observed for nalidixic acid (93/127; 73.2%), ampicillin (78/127; 61.4%), tetracycline (71/127; 55.9%), trimethoprim–sulfamethoxazole (43/127; 33.9%), amoxicillin–clavulanic acid (33/127; 26.0%), chloramphenicol (29/127; 22.8%), and ciprofloxacin (24/127; 18.9%). Lower rates were recorded for colistin by MIC (17/127; 13.4%), gentamicin (11.8%), cefepime (4.7%), cefotaxime (4.7%), amikacin (4.7%), ertapenem (3.1%), meropenem (3.1%), and cefoxitin (1.6%). No resistance was detected to imipenem or piperacillin–tazobactam.

Direct amoxicillin and azithromycin disk results were retained as raw screening data in Supplementary Table S2 but were excluded from categorical summaries, temporal models, and MDR classification because general CLSI M100 breakpoints were not applicable to these organism–agent combinations. *Salmonella* levofloxacin disk results were likewise not categorically interpreted. Nalidixic acid was retained as a phenotypic screening marker and was not used alone to infer clinical fluoroquinolone susceptibility. Complete isolate-level results, colistin MICs, interpretive criteria, and exclusions are provided in Supplementary Table S2.

### 2.5. Monthly Evolution of Antimicrobial Resistance in E. coli and Salmonella spp.

**Figure 4:**
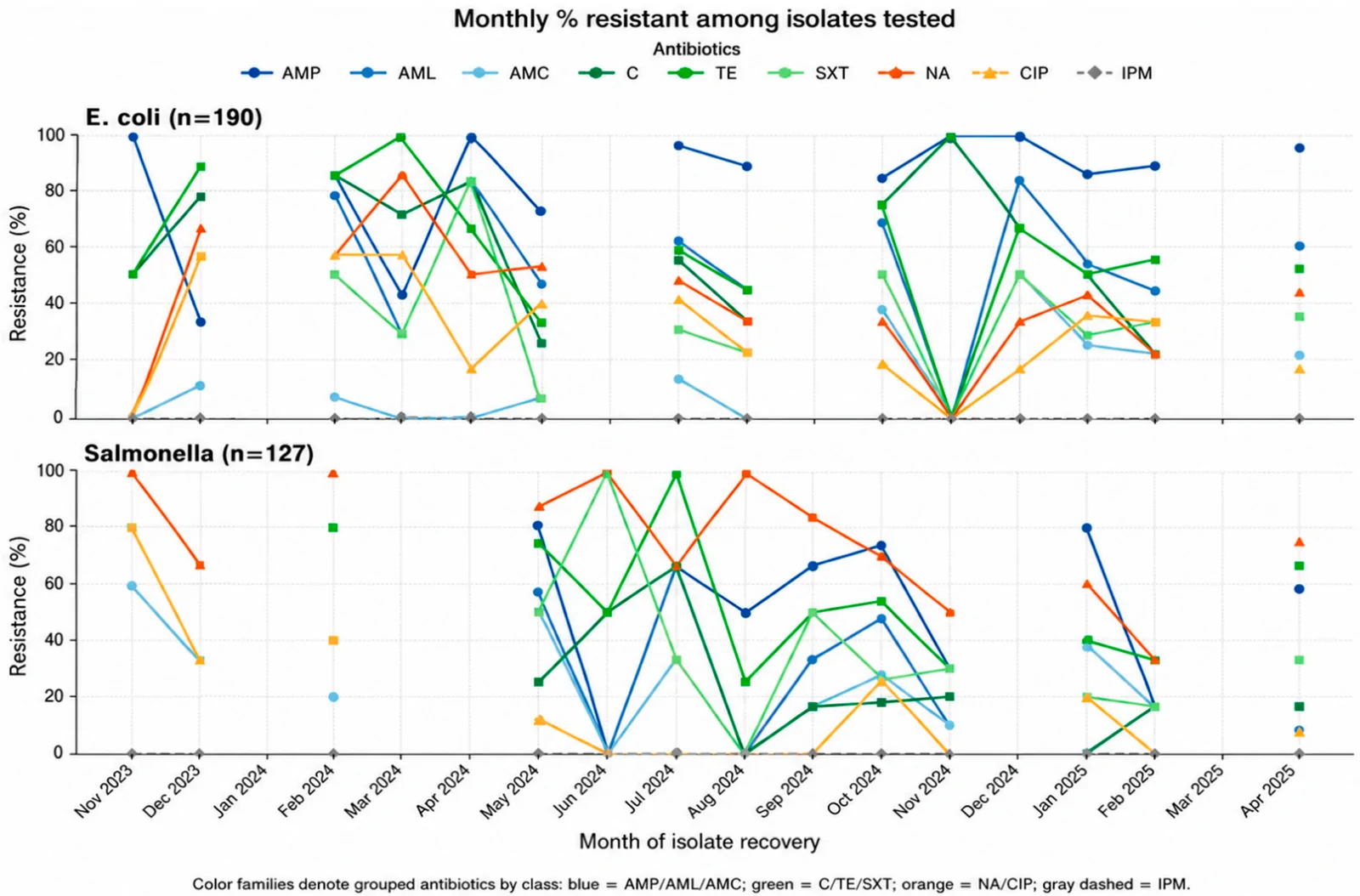
Descriptive monthly resistance percentages among *Escherichia coli* and *Salmonella* spp. isolates. The denominator for each monthly percentage is the number of isolates of the corresponding bacterial group recovered in that month. AMP: ampicillin; AMC: amoxicillin–clavulanic acid; C: chloramphenicol; TE: tetracycline; SXT: trimethoprim–sulfamethoxazole; NA: nalidixic acid; CIP: ciprofloxacin; IPM: imipenem. Monthly denominators are provided in Supplementary Table S1.

Monthly resistance percentages varied markedly in both bacterial groups (Figure 4). Because the numbers and environmental-matrix composition of isolates differed among months, the curves are descriptive and should not be interpreted as seasonality or as unadjusted evidence of a temporal increase or decrease. The number of isolates tested in each month is provided in Supplementary Table S1.

In *E. coli*, ampicillin resistance remained frequent across most sampled months, while tetracycline, chloramphenicol, trimethoprim–sulfamethoxazole, nalidixic acid, ciprofloxacin, and amoxicillin–clavulanic acid showed variable monthly percentages. No imipenem resistance was detected in any month. Months represented by few isolates produced unstable percentages and were interpreted cautiously.

In *Salmonella* spp., nalidixic acid, ampicillin, and tetracycline were the most consistently observed resistance phenotypes, whereas ciprofloxacin, chloramphenicol, trimethoprim–sulfamethoxazole, and amoxicillin–clavulanic acid varied across sampled months. No imipenem resistance was detected. These descriptive patterns were evaluated further only through models that adjusted for matrix and farm-level clustering.

Overall, Figure 4 illustrates repeated detection of resistance during the study period, not a balanced longitudinal time series. Adjusted temporal estimates are reported separately in Section 2.6 and should be interpreted together with the irregular sampling design and the month-level denominators in Supplementary Table S1.

### 2.6. Temporal Trends in Antimicrobial Resistance Based on Mixed-Effects Logistic Regression

Mixed-effects logistic regression adjusted for sampling matrix and included farm as a random intercept (Table 3). After Benjamini–Hochberg correction, increasing monthly associations remained statistically significant only for ampicillin resistance in *E. coli* (adjusted OR = 1.22, 95% CI: 1.09–1.35; q = 0.0033) and amoxicillin–clavulanic acid resistance in *E. coli* (adjusted OR = 1.18, 95% CI: 1.06–1.31; q = 0.0087). No temporal association remained significant for *Salmonella* spp. after correction. These estimates represent adjusted associations within an irregularly sampled dataset and should not be interpreted as proof of a population-level temporal trend or seasonality.

### 2.7. Multidrug Resistance Distribution and Predominant Resistance-Class Combinations

Among 190 *E. coli* isolates, 115 (60.5%) met the MDR definition (Table 4). The highest matrix-specific proportions were observed in farm soil (36/42; 85.7%), drinking troughs (20/24; 83.3%), and poultry manure (28/37; 75.7%). Lower proportions were observed in well water (10/22; 45.5%), soil approximately 200 m from the farms (10/30; 33.3%), and soil approximately 500 m from the farms (11/35; 31.4%).

Among 127 *Salmonella* spp. isolates, 82 (64.6%) were MDR. The highest proportions occurred in poultry manure (26/28; 92.9%) and drinking troughs (18/23; 78.3%), followed by well water (11/18; 61.1%), soil approximately 200 m from the farms (8/14; 57.1%), and farm soil (18/34; 52.9%). One of ten isolates from soil approximately 500 m from the farms was MDR (10.0%). These matrix-specific estimates describe the sampled isolates and do not demonstrate movement between matrices.

The predominant non-mutually exclusive three-class combination among MDR *E. coli* was β-lactams–tetracyclines–phenicols (85/115; 73.9%), followed by tetracyclines–phenicols–quinolones (69/115; 60.0%), β-lactams–tetracyclines–quinolones (67/115; 58.3%), and β-lactams–phenicols–quinolones (64/115; 55.7%) (Table 5). Combinations involving folate-pathway inhibitors were also common.

Among MDR *Salmonella* spp., β-lactams–tetracyclines–quinolones was the most frequent combination (48/82; 58.5%), followed by tetracyclines–quinolones–folate inhibitors (31/82; 37.8%), β-lactams–quinolones–folate inhibitors (29/82; 35.4%), and β-lactams–tetracyclines–folate inhibitors (28/82; 34.1%). An isolate resistant to more than three classes could contribute to several combinations. Complete isolate-level drug and class profiles are provided in Supplementary Table S2.

## 3. Discussion

This study provides baseline evidence of culture-confirmed, phenotypically antimicrobial-resistant *Escherichia coli* and *Salmonella* spp. in broiler-farm environmental matrices in Morocco [23,24,25]. *E. coli* and *Salmonella* spp. were detected in 35.0% and 23.4% of 543 samples, respectively, and 60.5% and 64.6% of the corresponding isolates were MDR. After adjustment for matrix, farm-level clustering, and multiple comparisons, only ampicillin and amoxicillin–clavulanic acid resistance in *E. coli* showed statistically significant positive monthly associations. No adjusted temporal association remained significant for *Salmonella* spp. The findings describe an environmental AMR burden but do not establish persistence, source attribution, or transmission.

Detection rates differed among matrices. *E. coli* was detected most frequently in poultry manure, soil approximately 500 m from the farms, and farm soil, whereas *Salmonella* spp. was most frequently detected in poultry manure, farm soil, and drinking troughs. Frequent recovery from manure and farm soil is compatible with fecal deposition and organic-material accumulation [26]. Detection in drinking troughs may reflect contact with birds or persistence within water-system biofilms [27]. However, matrix comparisons must be interpreted cautiously because recovery can be affected by sample composition, bacterial survival, farm practices, and unequal sampling intensity [28,29,30,31,32,33].

The dominant phenotypic resistance patterns involved ampicillin, tetracycline, chloramphenicol, and quinolone-class agents in *E. coli*, and nalidixic acid, ampicillin, and tetracycline in *Salmonella* spp. These profiles are broadly consistent with resistance reported in poultry-associated Enterobacterales [18,34,35,36,37,38]. Because farm-level antimicrobial-use histories were unavailable, resistance cannot be attributed to specific compounds. Resistance to extended-spectrum cephalosporins, aminoglycosides, carbapenems, and colistin was less frequent, but these clinically important phenotypes warrant continued surveillance.

MDR was common in both bacterial groups and was particularly frequent in manure, drinking troughs, and farm soil. The predominant three-class profiles were β-lactams–tetracyclines–phenicols in *E. coli* and β-lactams–tetracyclines–quinolones in *Salmonella* spp. These combinations demonstrate phenotypic co-resistance only. Without resistance-gene, plasmid, integron, or whole-genome data, the study cannot determine whether resistance determinants were genetically linked or shared between isolates [18,39].

From a One Health perspective, resistant bacteria detected in manure, farm soil, drinking systems, surrounding soils, and well water support inclusion of poultry-farm environments in AMR surveillance. These matrices may help identify practical intervention points, including manure and litter management, water-line sanitation, well protection, farm biosecurity, and responsible veterinary antimicrobial use [40,41,42,43,44]. The cross-sectional findings should nevertheless be regarded as phenotypic baseline surveillance rather than evidence of transmission.

Detection in soils approximately 200 and 500 m from poultry houses and in well water establishes the presence of resistant bacteria in external environmental matrices. Because strain typing, hydrological assessment, manure-management tracing, and environmental source tracking were not performed, neither the origin nor the direction of bacterial movement can be determined. Runoff, dust, insects, equipment, footwear, or manure transport remain possible hypotheses for future investigation; none was tested here. The results also do not demonstrate exposure or transmission to workers, residents, animals, groundwater, or the food chain.

Strengths of the study include the analysis of 543 samples from six matrices across 13 farms, simultaneous assessment of two relevant bacterial groups, real-time PCR confirmation of *Salmonella* spp., isolate-level AST reporting, MIC-based colistin testing, and mixed-effects models accounting for matrix and farm clustering. Limitations include purposive farm selection, irregular and unbalanced sampling, lack of bacterial concentration measurements, absence of farm antimicrobial-use and environmental covariates, and retention of one isolate per species per positive sample. *Salmonella* serotyping was not performed, and phenotypic testing could not identify resistance genes, plasmids, integrons, or genetic relationships. Future work should combine serotyping, whole-genome sequencing, plasmid analysis, hydrological assessment, and source-tracking methods.

## 4. Materials and Methods

### 4.1. Study Design, Study Area, and Farms

A cross-sectional field study was conducted in broiler farms in the Rabat–Salé–Kénitra region of Morocco [45]. Sampling occurred on 35 separate dates between 24 November 2023 and 28 April 2025. Intervals between consecutive field dates ranged from 1 to 63 days; some months included multiple visits and others had no sampling. The schedule was determined by farm accessibility, active broiler-production cycles, biosecurity requirements, and field logistics and was not intended to provide fixed monthly or seasonal coverage.

Thirteen farms were selected purposively from farms listed in official registries maintained by the Moroccan agricultural authorities, based on access and availability during the study period. The sample was therefore not a probability sample and should not be considered statistically representative of all farms in the region. All 13 farms were sampled repeatedly; farm totals ranged from 39 to 46 samples collected on 10 to 17 distinct dates. Sampling intensity was not balanced across farms, months, or matrices. Farm-by-matrix and month-by-matrix counts are provided in Supplementary Table S1. The farm locations are shown in Figure 2.

### 4.2. Sample Collection and Transport

Samples were collected from six matrices during broiler-production cycles: poultry manure (n = 78), drinking troughs (n = 79), farm soil (n = 98), soil approximately 200 m from the poultry houses (n = 79), soil approximately 500 m from the poultry houses (n = 79), and well water (n = 130), for a total of 543 samples. The same farms were visited repeatedly, and one or more matrices were collected according to availability at each visit. Multiple observations from a farm were therefore not assumed to be independent in the temporal models. Row-level sampling metadata and the complete farm/month/matrix distribution are provided in Supplementary Table S1. The study did not follow individual birds or specific flocks; repeated visits could occur during successive commercial broiler-production cycles. Flock diet and farm-level antimicrobial-use histories were not recorded.

Approximately 25 g of each solid matrix was collected aseptically into a sterile sampling bag. Well-water samples were collected in sterile containers. Samples were transported to the laboratory in cooled containers at approximately 4 °C and processed upon arrival.

### 4.3. Isolation and Identification of Escherichia coli

For solid samples, 25 g was homogenized with 225 mL of buffered peptone water and incubated at 37 °C for 16–24 h. Aliquots were streaked onto eosin methylene blue agar (Oxoid, Basingstoke, UK) and incubated aerobically at 37 °C for 24 h. Colonies with a characteristic green metallic sheen were considered presumptive *E. coli* and were subcultured on nutrient agar for purification.

For well water, 100 mL was filtered through a 0.45 µm membrane. The membrane was placed on eosin methylene blue agar and incubated aerobically at 37 °C for 24 h. Presumptive colonies were purified as described above.

Biochemical identification was performed with API 20E strips (bioMérieux, Marcy-l’Étoile, France) according to the manufacturer’s instructions. Strips were incubated at 36 ± 2 °C for 18–24 h, and numerical profiles were interpreted with the APIweb™ identification database (bioMérieux; https://apiweb.biomerieux.com; accessed on 18 July 2026). Doubtful or low-discrimination profiles were repeated before inclusion. Confirmed isolates were stored in 20% glycerol at −20 °C.

### 4.4. Isolation and Identification of Salmonella spp.

For solid samples, 25 g was homogenized with 225 mL of buffered peptone water and incubated at 37 °C for 16–24 h. A 0.1 mL aliquot was transferred to 10 mL of Rappaport–Vassiliadis enrichment broth and incubated at 42 °C for 18 h. Enriched cultures were streaked on xylose lysine deoxycholate agar and incubated at 36 °C for 18–24 h.

For well water, 1 L was filtered through a 0.45 µm membrane. The membrane was transferred to Rappaport–Vassiliadis enrichment broth and incubated at 42 °C for 18 h, after which aliquots were streaked on xylose lysine deoxycholate agar and incubated at 36 °C for 18–24 h.

Presumptive *Salmonella* colonies were purified on nutrient agar and identified biochemically using API 20E strips according to the manufacturer’s instructions. Profiles were interpreted with APIweb after incubation at 36 ± 2 °C for 18–24 h, and doubtful or low-discrimination profiles were repeated. Biochemically identified isolates were stored in 20% glycerol at −20 °C pending molecular confirmation.

### 4.5. Molecular Confirmation of Salmonella spp.

All biochemically identified *Salmonella* spp. isolates were confirmed by real-time PCR. Genomic DNA was extracted from pure-culture bacterial suspensions with the Maxwell^®^ RSC PureFood Pathogen Kit (Promega, Madison, WI, USA), using the manufacturer’s Quick Protocol with the bacterial suspension substituted for the food homogenate. The innuDETECT™ *Salmonella* spp. Assay (Analytik Jena, Jena, Germany), a TaqMan-based qualitative assay, was run on a QuantStudio™ 5 Real-Time PCR System (Applied Biosystems, Thermo Fisher Scientific, Waltham, MA, USA) according to the manufacturer’s instructions. Positive and negative controls were included in each run. Serovar identification was not performed.

### 4.6. Antimicrobial Susceptibility Testing

One confirmed isolate per bacterial species and per positive environmental sample was retained for antimicrobial susceptibility testing. When several colonies with the same presumptive morphology were present, one purified representative colony was selected. Consequently, 190 *E. coli* and 127 *Salmonella* spp. isolates were tested, and within-sample strain diversity was not assessed.

Disk diffusion was performed on Mueller–Hinton agar (Oxoid, Basingstoke, UK) using a 0.5 McFarland suspension prepared from fresh colonies. Plates were inoculated uniformly, disks were applied, and plates were incubated at 35 ± 2 °C for 16–18 h unless otherwise specified in the applicable table. Zone diameters were measured in millimeters and interpreted using the organism-specific criteria in CLSI M100, 31st edition (2021), Table S2A and associated *Salmonella* footnotes [46]. *E. coli* ATCC 25922, the routine CLSI quality-control strain for Enterobacterales, was used to monitor disk-diffusion performance for both *E. coli* and *Salmonella* spp.

The categorically interpreted disks were ampicillin (10 µg), amoxicillin–clavulanic acid (20/10 µg), cefoxitin (30 µg), cefotaxime (30 µg), cefepime (30 µg), piperacillin–tazobactam (100/10 µg), gentamicin (10 µg), amikacin (30 µg), nalidixic acid (30 µg), ciprofloxacin (5 µg), levofloxacin (5 µg; *E. coli* only), trimethoprim–sulfamethoxazole (1.25/23.75 µg), chloramphenicol (30 µg), tetracycline (30 µg), imipenem (10 µg), ertapenem (10 µg), and meropenem (10 µg). Direct amoxicillin (25 µg) and azithromycin (15 µg) results were retained as raw screening observations but excluded from categorical analysis because general CLSI criteria were not applicable. *Salmonella* levofloxacin disk results were also excluded. Exact susceptible, intermediate or susceptible-dose-dependent, and resistant zone criteria are listed in Supplementary Table S2.

Colistin was not assessed by disk diffusion. MICs were determined for all 317 isolates using the CLSI colistin broth disk elution method [46]. Four tubes containing 10 mL of cation-adjusted Mueller–Hinton broth were prepared as a growth control and at final colistin concentrations of 1, 2, and 4 µg/mL using 10 µg colistin disks. After 30 min of elution at room temperature, 50 µL of a 0.5 McFarland suspension was added to each tube, producing an approximate final inoculum of 7.5 × 10^5^ CFU/mL. Tubes were incubated at 35 ± 2 °C for 16–20 h, and the MIC was the lowest concentration with complete inhibition of visible growth. For Enterobacterales, MICs ≤ 2 µg/mL were categorized as intermediate and MICs ≥4 µg/mL as resistant; CLSI provides no susceptible category. Isolate-level growth patterns and MICs are reported in Supplementary Table S2.

### 4.7. Definition of Multidrug Resistance

MDR was defined as resistance to at least one antimicrobial agent in three or more antimicrobial classes [47]. Intermediate and susceptible-dose-dependent results were coded as non-resistant. Only agents with applicable categorical interpretation criteria were used for MDR classification; raw amoxicillin and azithromycin screens and *Salmonella* levofloxacin disk results were excluded. Each antimicrobial class was counted once per isolate.

### 4.8. Statistical Analysis

Data were summarized by bacterial species, sampling matrix, farm, location, date, and AST profile. Detection rates were calculated as positive samples divided by samples analyzed for each bacterial group and matrix. Resistance percentages were calculated as resistant isolates divided by isolates tested and categorically interpretable for the corresponding agent. Farm- and month-level sample distributions and isolate denominators are provided in Supplementary Table S1.

Temporal associations were evaluated separately for *E. coli* and *Salmonella* spp. by mixed-effects logistic regression. Resistance was the binary outcome (resistant = 1; susceptible, intermediate, or susceptible-dose-dependent = 0), and calendar time was coded as months since the first sampling date. Sampling matrix was included as a categorical fixed effect and farm as a random intercept to account for repeated sampling and within-farm clustering. The primary model was resistance ~ month + matrix + (1|farm).

A sampling visit was defined as a farm–date combination. A visit-level random intercept was examined in sensitivity analyses but was not retained when its variance approached zero, convergence was unstable, or model fit was not meaningfully improved. Models were fitted only when at least 10 resistant and 10 non-resistant isolates were available and were not interpreted when complete or quasi-complete separation produced unstable estimates.

Adjusted odds ratios and 95% confidence intervals estimated the association with a one-month increase after adjustment for matrix and farm clustering. Benjamini–Hochberg false-discovery-rate correction was applied separately by species, with q < 0.05 considered statistically significant. Because sampling was irregular and unbalanced, descriptive monthly percentages and adjusted month coefficients were not interpreted as seasonality, causal change, or population-level trends. Analyses were performed in R (R Foundation for Statistical Computing, Vienna, Austria; https://www.r-project.org/; accessed on 18 July 2026) using the lme4 package and the supporting packages broom.mixed, dplyr, tidyr, purrr, and readxl.

## 5. Conclusions

This phenotypic baseline study detected antimicrobial-resistant *E. coli* and *Salmonella* spp. across multiple broiler-farm environmental matrices in Morocco, with a substantial MDR burden in both bacterial groups. Broiler-farm matrices may act as environmental reservoirs and should be incorporated into One Health surveillance and farm-level control programs. However, detection in surrounding soils and well water does not establish origin, persistence, or direction of spread. Serotyping, whole-genome sequencing, hydrological assessment, and source-tracking studies are needed to resolve transmission pathways.

## Figures and Tables

**Table 1:** Detection rates of *Escherichia coli* and *Salmonella* spp. according to environmental matrix in broiler farms.

Matrix/Source	*E. coli Positive,* n	*E. coli Detection Rate* (%)	*Salmonella* spp. *Positive,* n	*Salmonella Detection Rate* (%)	Samples Analyzed, n
Poultry manure	37	47.4	28	35.9	78
Well water	22	16.9	18	13.8	130
Soil	42	42.9	34	34.7	98
Soil around 200 m	30	38.0	14	17.7	79
Soil around 500 m	35	44.3	10	12.7	79
Drinking trough	24	30.4	23	29.1	79
**Total**	**190**	**35.0**	**127**	**23.4**	**543**

Bold indicates the total summary row.

**Table 2:** Geographical distribution of *E. coli* and *Salmonella* spp. detections according to study location.

Location	*E. coli* Detected, n (%)	*Salmonella* spp. Detected, n (%)
Sidi Yahia	61 (32.1)	37 (29.1)
Mers El Kheir	45 (23.7)	30 (23.6)
Sala Jadida	45 (23.7)	29 (22.8)
Shoul	39 (20.5)	31 (24.4)
**Total**	**190 (100)**	**127 (100)**

Percentages represent the proportion of total positive detections for each bacterial group. Bold indicates the total summary row.

**Table 3:** Adjusted monthly associations with antimicrobial resistance estimated by mixed-effects logistic regression.

Species	Antibiotic	Resistant Isolates, n/N (%)	Adjusted OR Per Month (95% CI)	Raw *p*-Value	BH-FDR q-Value
*E. coli*	*Ampicillin (AMP)*	161/190 (84.7%)	**1.22 (1.09–1.35)**	0.0003	**0.0033**
*E. coli*	*Nalidixic acid (NA)*	85/190 (44.7%)	0.96 (0.90–1.02)	0.1927	0.2630
*E. coli*	*Trimethoprim–sulfamethoxazole (SXT)*	69/190 (36.3%)	1.02 (0.95–1.09)	0.6608	0.7342
*E. coli*	*Amoxicillin (AML)*	Raw screen only	—	—	—
*E. coli*	*Amoxicillin–clavulanic acid (AMC)*	36/190 (18.9%)	**1.18 (1.06–1.31)**	0.0017	**0.0087**
*E. coli*	*Levofloxacin (LEV)*	47/190 (24.7%)	1.00 (0.93–1.07)	0.9783	0.9783
*E. coli*	*Ciprofloxacin (CIP)*	62/190 (32.6%)	0.93 (0.86–1.00)	0.0417	0.1041
*E. coli*	*Chloramphenicol (C)*	106/190 (55.8%)	0.92 (0.85–1.00)	0.0568	0.1135
*E. coli*	*Tetracycline (TE)*	117/190 (61.6%)	0.95 (0.88–1.03)	0.2104	0.2630
*E. coli*	*Azithromycin (AZM)*	Raw screen only	—	—	—
*Salmonella* spp.	Ampicillin (AMP)	78/127 (61.4%)	1.07 (0.96–1.18)	0.2191	0.2921
*Salmonella* spp.	Nalidixic acid (NA)	93/127 (73.2%)	0.90 (0.79–1.03)	0.1171	0.2341
*Salmonella* spp.	Trimethoprim–sulfamethoxazole (SXT)	43/127 (33.9%)	0.88 (0.79–0.98)	0.0249	0.0995
*Salmonella* spp.	Tetracycline (TE)	71/127 (55.9%)	0.95 (0.85–1.06)	0.3744	0.3744

Model: resistance ~ month + matrix + (1 | farm). Resistant isolates were coded 1 and susceptible, intermediate, or susceptible-dose-dependent results were coded 0. Odds ratios describe the adjusted association with a one-month increase. Benjamini–Hochberg correction was applied separately by species; bold values indicate q < 0.05. Models with sparse outcomes, separation, or unstable estimates were excluded. Amoxicillin and azithromycin screening results were not modeled because applicable general CLSI M100 categorical criteria were unavailable.

**Table 4:** Distribution of multidrug-resistant *E. coli* and *Salmonella* spp. isolates according to environmental matrix.

Matrix/Source	*E. coli* Positive Isolates	MDR *E. coli*, n (%)	*Salmonella* Positive Isolates	MDR *Salmonella* spp., n (%)	Samples Analyzed
Poultry manure	37	28 (75.7)	28	26 (92.9)	78
Drinking trough	24	20 (83.3)	23	18 (78.3)	79
Soil	42	36 (85.7)	34	18 (52.9)	98
Soil around 200 m	30	10 (33.3)	14	8 (57.1)	79
Soil around 500 m	35	11 (31.4)	10	1 (10.0)	79
Well water	22	10 (45.5)	18	11 (61.1)	130
**Overall**	**190**	**115 (60.5)**	**127**	**82 (64.6)**	**543**

Percentages were calculated among positive isolates of the corresponding bacterial group within each matrix. MDR classification used only antimicrobial agents with applicable categorical interpretation criteria. Bold indicates the overall summary row.

**Table 5:** Most frequent three-class multidrug-resistance combinations among *Escherichia coli* and *Salmonella* spp. isolates.

Species	MDR Class Combination	Number	Percentage of MDR Isolates
*E. coli*	*β-lactams–tetracyclines–phenicols*	85	73.9%
*E. coli*	*tetracyclines–phenicols–quinolones*	69	60.0%
*E. coli*	*β-lactams–tetracyclines–quinolones*	67	58.3%
*E. coli*	*β-lactams–phenicols–quinolones*	64	55.7%
*E. coli*	*β-lactams–tetracyclines–folate inhibitors*	61	53.0%
*E. coli*	*tetracyclines–phenicols–folate inhibitors*	60	52.2%
*E. coli*	*β-lactams–phenicols–folate inhibitors*	60	52.2%
*Salmonella* spp.	β-lactams–tetracyclines–quinolones	48	58.5%
*Salmonella* spp.	tetracyclines–quinolones–folate inhibitors	31	37.8%
*Salmonella* spp.	β-lactams–quinolones–folate inhibitors	29	35.4%
*Salmonella* spp.	β-lactams–tetracyclines–folate inhibitors	28	34.1%
*Salmonella* spp.	tetracyclines–phenicols–folate inhibitors	21	25.6%
*Salmonella* spp.	phenicols–quinolones–folate inhibitors	20	24.4%

Values are the number and percentage of MDR isolates resistant to at least one agent in each indicated class. Denominators were 115 MDR *E. coli* isolates and 82 MDR *Salmonella* spp. isolates. Combinations were non-mutually exclusive and therefore do not sum to 100%. Full isolate-level profiles are provided in Supplementary Table S2.

## Data Availability

The data presented in this study are available in the article and its Supplementary Materials.

## Supplementary Materials

The following supporting information can be downloaded at: https://www.mdpi.com/article/10.3390/antibiotics15080731/s1.

## Abbreviations

The following abbreviations are used in this manuscript:

**Abbreviation**	**Full form**
AK	Amikacin
AMC	Amoxicillin–clavulanic acid
AML	Amoxicillin
AMR	Antimicrobial resistance
AMP	Ampicillin
API 20E	Analytical Profile Index 20E
ARGs	Antimicrobial resistance genes
AST	Antimicrobial susceptibility testing
ATCC	American Type Culture Collection
AZM	Azithromycin
BPW	Buffered peptone water
C	Chloramphenicol
CI	Confidence interval
CIP	Ciprofloxacin
CLSI	Clinical and Laboratory Standards Institute
CN	Gentamicin
COL	Colistin
CTX	Cefotaxime
*E. coli*	*Escherichia coli*
EMB	Eosin methylene blue agar
ETP	Ertapenem
FDR	False discovery rate
FEP	Cefepime
FOX	Cefoxitin
IPM	Imipenem
LEV	Levofloxacin
MDR	Multidrug resistance/multidrug-resistant
MEM	Meropenem
MIC	Minimum inhibitory concentration
NA	Nalidixic acid
OR	Odds ratio
spp.	Species plural
SXT	Trimethoprim–sulfamethoxazole
TE	Tetracycline
TZP	Piperacillin–tazobactam
WOAH	World Organization for Animal Health
XLD	Xylose lysine deoxycholate agar
